# Genotype-Environment Interaction in ADHD: Genetic Predisposition Determines the Extent to Which Environmental Influences Explain Variability in the Symptom Dimensions Hyperactivity and Inattention

**DOI:** 10.1007/s10519-023-10168-5

**Published:** 2024-01-25

**Authors:** Inga Schwabe, Miljan Jović, Kaili Rimfeld, Andrea G. Allegrini, Stéphanie M. van den Berg

**Affiliations:** 1 Department of Methodology and Statistics, Tilburg University, Tilburg, The Netherlands; 2 Department of Cognition, Data and Education (CODE), University of Twente, Enschede, The Netherlands; 3 Social, Genetic and Developmental Psychiatry Centre, Institute of Psychiatry, Psychology and Neuroscience, King’s College London, London, UK; 4 Department of Psychology, Royal Holloway, University of London, Egham, UK; 5 Division of Psychology and Language Sciences, Department of Clinical, Educational and Health Psychology, University College London, London, UK

**Keywords:** ADHD, Hyperactivity, Inattention, Genotype-environment interaction, IRT, Twin study, Measurement error, Environmental influences

## Abstract

Although earlier research has shown that individual differences on the spectrum of attention deficit hyperactivity disorder (ADHD) are highly heritable, emerging evidence suggests that symptoms are associated with complex interactions between genes and environmental influences. This study investigated whether a genetic predisposition [Note that the term ‘genetic predisposition’ was used in this manuscript to refer to an estimate based on twin modeling (an individual’s score on the latent trait that resembles additive genetic influences) in the particular population being examined.] for the symptom dimensions *hyperactivity* and *inattention* determines the extent to which unique-environmental influences explain variability in these symptoms. To this purpose, we analysed a sample drawn from the Twins Early Development Study (TEDS) that consisted of item-level scores of 2168 16-year-old twin pairs who completed both the *Strengths and Difficulties Questionnaire* (SDQ; Goodman, in J Child Psychol Psychiatry 38:581–586, 1997) and the *Strength and Weaknesses of ADHD Symptoms and Normal Behavior* (SWAN; Swanson, in Paper presented at the meeting of the American Psychological Association, Los Angeles, 1981) questionnaire. To maximize the psychometric information to measure ADHD symptoms, psychometric analyses were performed to investigate whether the items from the two questionnaires could be combined to form two longer subscales. In the estimation of genotype-environment interaction, we corrected for error variance heterogeneity in the measurement of ADHD symptoms through the application of item response theory (IRT) measurement models. A positive interaction was found for both hyperactivity (e.g., ${\beta }_{1}$ = 2.20 with 95% highest posterior density interval equal to [1.79;2.65] and effect size equal to 3.00) and inattention (e.g., ${\beta }_{1}$ = 2.16 with 95% highest posterior density interval equal to [1.56;2.79] and effect size equal to 3.07). These results indicate that unique-environmental influences were more important in creating individual differences in both hyperactivity and inattention for twins with a genetic predisposition for these symptoms than for twins without such a predisposition.

**Supplementary Information:**

The online version contains supplementary material available at 10.1007/s10519-023-10168-5.

## Introduction

Attention deficit hyperactivity disorder (ADHD) affects roughly 3.4% of the worldwide child and adolescent population (Polanczyk et al. 2015). Symptoms of ADHD are measured on two different core dimensions, *hyperactivity* and *inattention*, which form the basis of the current *Diagnostic and Statistical Manual of Mental Disorders* subtype classification system. Depending on the (combination of) symptoms, individuals with ADHD are easily distracted during tasks and conversations, experience severe difficulty in sustaining activities, or show a deficit in inhibitory control (Scheres et al. 2003). This can cause difficulties with school education, lead to emotional and behavioural difficulties or impair peer relationships. For 50% of the ADHD patient population, symptoms persist into adulthood (Schmitz et al. 2007) and may have a significant impact on academic and personal life, due to, for example, poorer academic development, family problems or higher health care expenses (Coghill et al. 2008).

With heritability estimates ranging from 70 to 90% for both symptom dimensions, the results of genetically-informative twin studies have consistently demonstrated that variation in the two core dimensions of ADHD is strongly influenced by genetic factors (e.g., Willcutt 2005; Rietveld et al. 2004; Hudziak et al. 2000; Faraone and Doyle 2002; Faraone and Larsson 2019). A comprehensive meta-analysis by Nikolas and Burt (2010) that was based on the results of 79 twin and adoption studies showed that, while both dimensions are highly heritable, hyperactivity and inattention are distinct as to the *amount* of additive and dominant genetic influences that are important in creating individual differences: Nikolas and Burt (2010) showed that variance within the hyperactivity dimension could be mainly explained by additive genetic influences (71%) while dominant genetic influences contributed only a small proportion to the variance (2%). For inattention, additive genetic influences were also the most important source of individual differences, but the amount was smaller compared to hyperactivity (56%), while the contribution of dominant genetic influences was larger (15%). Furthermore, the remaining variance (27% for hyperactivity and 29% for inattention) could be mostly explained by environmental influences that are unique to a single twin (i.e., unique-environmental influences). That is, the shared environment did not account for a significant proportion of individual differences, a result replicated in many other studies. The authors of the meta-analysis furthermore found that results were consistent among several moderating factors, including gender, age, and measurement instrument. Note that while the meta-analysis by Nikolas and Burt (2010) suggests the presence of dominant genetic influences, other research has highlighted that the observed correlation patterns among monozygotic (MZ) and dizygotic (DZ) might more likely be explained by the presence of sibling contrast effects: Parents may either stress the similarities or the differences between their children which can result in cooperation or competition within a twin pair (influencing the degree of hyperactivity or inattention). Research in ADHD has consistently found evidence for the presence of this effect (see Rietveld et al. 2003). However, note that in the case that both mechanisms act on ADHD, statistical power is very low to also detect additive genetic influences (see Rietveld et al. 2003 for power calculations).

While genetic influences are strongly implicated in the aetiology of ADHD, it remains difficult to characterize the *particular* environmental circumstances under which ADHD symptoms emerge. The reason for this difficulty might lie in the complex nature of the condition: Similar to other neurodevelopmental disorders, ADHD likely results from the combined action of multiple genes with small effects and various environmental risk factors. Additionally, a simple distinction between nature, on the one hand, and nurture, on the other hand, might be too simplistic because there is emerging evidence that complex traits are associated with interactions between genetic- and environmental influences. In this manuscript, we focus on the latter: How do genes and environmental stressors *interact* to create individual differences on the ADHD spectrum?

### Genotype-environment interaction

A *genotype-environment interaction* formally refers to a situation in which the relative importance of environmental influences in explaining individual differences in a trait is conditional on the genotype (or vice versa). Applied to ADHD, this could, for example, mean that environmental influences are more important in creating individual differences in children with a genetic predisposition towards ADHD than for children without such a predisposition. Various studies suggest that genotype-environment interaction is an important phenomenon in mental disorders (see e.g., Wermter et al. 2010). Yet, research on genotype-environment interaction has not been a prominent focus: Most previous studies on genotype-environment interaction in ADHD have examined interactions between environments and candidate genes (with known limitations, see e.g., Munafo et al. 2014) while only a few studies focused on twin modelling. In one of the, to our knowledge, few published papers, Gould et al. (2018) investigated whether there is an interaction between additive genetic effects on ADHD symptoms and two specific environmental factors: socioeconomic status (SES) and chaos (household disorganization). Based on a population sample of 520 twin pairs, results showed that neither SES nor chaos was associated with a change in the extent to which genetic influences explain variability in ADHD symptoms. Note, however, that a plausible source for these non-findings might be insufficient power—earlier research has shown that much larger sample sizes are needed to detect genotype-environment interaction when using the method that was used by Gould et al. (2018) (Hanscombe et al. 2012). Furthermore, a Swedish population-based twin study used the data of 1518 twin pairs to explore the effects of dietary factors and found that genetic influences on inattention symptoms were statistically higher among twins with higher levels of high-sugar and unhealthy food intake compared to those with lower levels of high-sugar and unhealthy food consumption (Li et al. 2022).

Gould et al. (2018) and Li et al. (2022) tested genotype-environment interaction in the case that environmental influences are *specific*, measured variables. They tested two variables, but there are many possible environmental factors that could interact with additive genetic effects. Instead of testing them all separately, in the present study, we applied an *omnibus* test to assess whether there is *any* statistically significant interaction between genetic influences and unique-environmental influences. Thus, instead of focusing on one (or more) measured environmental stressors, this method parametrizes environmental influences as one (unobserved) latent variable, and we then test if this latent variable interacts with genetic effects. As a result, we focus on the whole range of possible (relevant) environmental factors (summarized in one latent variable), resulting in much larger power compared to focusing on one or more specific environmental stressors (see Schwabe and van den Berg 2014). This results in proper statistical power to determine—in an exploratory way—whether there is *any* genotype-environment interaction at all. If indeed an interaction effect is found, future research on the aetiology of ADHD can focus on the exact nature of this effect by collecting *specific* unique-environmental measures. Unlike Gould et al. (2018), we focus specifically on *unique*-environmental influences, since earlier results showed that shared environmental influences contribute no variance in ADHD, making it unlikely to find an interaction between genetic effects and shared environmental factors.

### Psychometric issues in the diagnosis of ADHD symptoms

Measurement of ADHD based on questionnaire data typically involves the construction of a composite score, such as a summation of endorsed symptoms or adding up a twin’s individual answers to all questionnaire items to form a sum score. However, applying this approach comes with a number of disadvantages in terms of methodological consequences and clinical interpretation. By calculating a composite score, all individual symptoms are weighted equally. Consequently, if one symptom is more indicative of the underlying dimension of ADHD than other symptoms, this is ignored. Furthermore, the use of composite scores neglects the uncertainty (e.g., the measurement error) that results from using only a limited set of items. This leads to a confounded measure of the underlying latent (i.e., not directly observable) trait, which can result not only in biased heritability estimates (van den Berg et al. 2007), but also in the spurious finding of a genotype-environment interaction effect. The latter has to do with the fact that the amount of information obtained from a questionnaire typically varies for different levels of the measured trait (see e.g. Loehlin and Nichols, 1976 and Turkheimer and Waldron, 2000). For example, while existing IQ tests usually show little measurement error for average students, scale scores can be very unreliable for high-performing students because there is only little information provided by only a few very difficult items. This is also the case for clinical scales: If both affected and healthy individuals with ADHD are assessed with a scale that contains many extreme items (for example: “I always have a hard time to pay attention”), then scale scores may be very reliable for twins that show ADHD symptoms, but very unreliable for healthy twins. In extreme situations (such as the ones just discussed), this often leads to floor or ceiling effects. A floor (ceiling) effect represents smaller individual differences at the lower (higher) end of the measurement scale. With other words: Measurement error is not homogeneous. This heterogeneity in measurement error leads to a skewed distribution of composite scores (e.g., sum scores). Research shows that this statistical artifact can result in the finding of a spurious genotype-environment interaction effect. For example, when our sample consists of high-school students and the conducted mathematics test is too easy for the most able twins, this will result in smaller score differences within highly able twin pairs than within average or less able twins (a ceiling effect). As a result, twins with a higher sum score seem more alike. In the case of a clinical scale, most healthy twins might not endorse extreme items which will result in smaller score differences within healthy twin pairs than within moderately ill or very ill twins (a floor effect). As a result, twins with a lower sum score seem more alike. In both scenarios, the finding of a spurious GxE effect can be expected—a positive effect in case of a ceiling effect and a negative effect in case of the floor effect (see e.g. Eaves et al. 1977; van der Sluis et al. 2006; Schwabe and van den Berg 2014; Molenaar and Dolan 2014).

Using simulation studies, Schwabe and van den Berg (2014; see also Molenaar and Dolan 2014) showed that this bias can be prevented by analyzing raw item scores, which can be done by simultaneously estimating the genetic twin model with an item response theory (IRT) model. Instead of ignoring measurement error as is done when using a composite score (like a sum score), the genetic variance decomposition (including genotype-environment interaction) is done directly on the latent variable that is corrected for measurement error. Crucial to this approach is that the analysis takes place in one unified model (estimating IRT and genetic model at the same time). When a so-called two-step procedure (i.e., using a measurement model to estimate latent trait scores and using these scores in a genetic design in a second step), the uncertainty of the measurement of the trait is not fully taken into account, and we can still expect spurious GxE.

### This research

We explored genotype-environment interaction in 2168 16-year-old twins who completed both the *Strengths and Difficulties Questionnaire* (SDQ; Goodman 1997) and the *Strength and Weaknesses of ADHD Symptoms and Normal Behavior* (SWAN; Swanson, 1981) questionnaire. To maximize the psychometric information available to measure ADHD symptoms, psychometric analyses were performed first to investigate whether the items from the respective subscales can be combined to form one more reliable scale. Next, genotype-environment interaction was studied separately for the two dimensions *hyperactivity* and *inattention,* applying the methodology from Schwabe and van den Berg (2014). This method corrects for error variance heterogeneity in the measurement of ADHD symptoms. As a result, our results regarding genotype-environment interaction as well as heritability estimates are free of the mentioned statistical artifacts due to measurement error (a limited number of questionnaire items) while at the same time, statistical power to detect an effect remains good (see Schwabe and van den Berg 2014 for technical details and power study).

## Method

### Sample

The data originate from the Twins Early Development Study (TEDS, Rimfeld et al. 2019), which is a longitudinal twin study that recruited twin pairs born between 1994 and 1996 in England and Wales through national birth records. From the total sample of twins that had answered the ADHD questionnaires, *N* = 656 individual twins were excluded from the analysis. *N* = 340 individual twins were excluded due to medical reasons, *N* = 168 because they were classified prenatal outliers, *N* = 64 due to unknown sex or zygosity, *N* = 28 due to the absence of first contact data, and for *N* = 56 multiple of the mentioned exclusion criteria applied (i.e., for *N* = 32 individual twins first contact data was absent and sex or zygosity was unknown, *N* = 22 were classified as both medical reason exclusion as a prenatal outlier and *N* = 2 were excluded due to medical reasons and unknown sex or zygosity).

All twin pairs excluded from analysis were from the same twin families (e.g., both first and co-twin had to be excluded), leading to a total *N* of 5517 twin pairs (N = 11,034 individual twins), of which 1991 monozygotic (MZ) twin pairs and 3526 dizygotic (DZ) twin pairs. Of the MZ pairs, 858 twin pairs were male, and 1133 were female. Of the DZ twin pairs, 796 pairs were male, 994 pairs were female and 1736 were opposite-sex twin pairs. Mean age of the twins at the time of return of the questionnaires was 16 years and three months (SD = 0.75, minimum age = 14 years and 9 months and maximum age = 18 years and 7 months). For the majority of the twins (*N* = 5293 twin pairs), English was the first language spoken at home and their ethnic origin was white (*N* = 5142 twin pairs).

To avoid bias in the psychometric analyses, due to the dependency in the data (e.g., twins being nested within families), we randomly selected one twin from every family for psychometric analyses. Missing data was treated by applying full information maximum likelihood (FIML), an approach to dealing with missing data that tends to yield equivalent results as multiple imputation (see e.g., Lee and Shi 2021).

Based on the results of the psychometric analyses, it was decided which subset of the data was used for genetic analyses (either only the items originating from the longer SWAN subscale or the combined scale based on both items from the SWAN and SDQ). Of this subset, data of families with missing data on all items were excluded from the analysis. This resulted in a sample size of *N* = 1084 twin pairs (415 MZ twin pairs and 669 DZ twin pairs) for both the hyperactivity and the inattention scale. Out of the remaining twin families, *N* = 1033 (95%) twin families had no missing data on the hyperactivity scale, within *N* = 20 (2%) families a single item answer was missing, *N* = 8 (1%) families missed 8 or fewer item scores and *N* = 23 (2%) had 9 unknown item scores. Concerning the inattention scale data, for *N* = 1016 (94%) twin pairs all item data were complete, within *N* = 34 (3%) families a single item answer was unknown, *N* = 11 (1%) twin pairs were missing 2 or up to 7 item scores and *N* = 23 (2%) families were missing 9 (*N* = 22 families) or 10 (*N* = 1 twin family) item scores.

### ADHD scales

The Strengths and Difficulties Questionnaire (SDQ; Goodman 1997) is a brief questionnaire for children and young people, meant for emotional and behavioural screening. With a total of 25 items, the hyperactivity/impulsive subscale of the questionnaire consists of 5 items and uses a three-point scale (0—*not true*, 1—*quite true* and 2—*very true*). Three of the items of this subscale measure inattention and two items hyperactivity. The items of the SDQ were part of the test booklet that was sent to twins at the age of 16 and answered by themselves (not their parents or teachers).

Developed by Swanson et al. (1981), the *Strength and Weaknesses of ADHD symptoms and Normal Behavior* (SWAN) questionnaire consists of two subscales of each 9 items, derived from the DSM-IV-TR (American Psychiatric Association 2008). The two subscales measure inattention and hyperactivity respectively using a seven-point scale of behaviour (1—*far below average*, 2—*below average*, 3—*slightly below average*, 4—*average*, 5—*slightly above average*, 6—*above average* and 7—*far above average*). Like the items from the SDQ, the items of the SWAN were part of the test booklet that was sent to twins at the age of 16 and answered by themselves (not their parents or teachers). Online Appendix A contains a list with all items included in the hyperactivity and inattention subscales of the SWAN and SDQ.

### Psychometric analyses

Psychometric analyses were conducted separately for hyperactivity (e.g., 9 items from the SWAN questionnaire and 2 items from the SDQ questionnaire) and for inattention (e.g., 9 items from the SWAN questionnaire and 3 items from the SDQ questionnaire). To investigate whether the two questionnaires measure the same underlying trait, we formed two long scales—consisting of a total of 11 items (hyperactivity) and 12 items (inattention) respectively. The item scores of the two combined scales were recoded such that a higher score on the combined scale reflected a higher trait score (i.e., a high degree of hyperactivity and inattention respectively).

To test whether combining the items from the different scales resulted in one unidimensional scale that measures the same latent trait (hyperactivity and inattention, respectively), multiple IRT models were estimated (e.g., the partial credit model (PCM), the generalized partial credit model (GPCM) and the graded response model (GRM)). Based on the lowest Akaike Information Criterion (AIC), the best-fitting IRT model was selected and used to investigate item information curves and item fit of the combined scales. For the estimation of all models, the R package mirt (Chalmers 2012) was used.

### Genetic models

Different genetic models were estimated. First of all, we used the ACE model that decomposes the phenotypic variance, ${\sigma }_{P}^{2}$, into variance that can be explained by additive genetic influences (e.g., ${\sigma }_{A}^{2}$), common-environmental influences (e.g., ${\sigma }_{C}^{2}$—parametrized as perfectly correlated within the same family) and unique-environmental influences (e.g., ${\sigma }_{E}^{2}$—parametrized to be correlated zero within one family). We furthermore considered an AE model (setting the C component to zero) and an ADE model in which the D component represents dominance effects (e.g., non-additive genetic variance).^1^

#### Genotype-environment interaction

In case of genotype-environment interaction, the amount of variance due to unique-environmental influences is not the same for every twin but depends on an individual’s genetic value, which is parametrized as a latent (e.g., unobserved) variable. This means that the unique-environmental variance component (e.g., ${\sigma }_{E}^{2}$) can be larger at either higher or lower levels of the genetic value. For this particular application, this means that unique-environmental influences are either more important for twins with a high genetic predisposition for ADHD (i.e., positive genotype-environment interaction) or for twins with no or only a weak genetic predisposition (i.e., negative genotype-environment interaction). A positive genotype-environment interaction predicts that twins with the same high genetic value (genetically predisposed for ADHD) are less similar (more variance) than twins with the same low genetic value (low predisposition for ADHD). A negative genotype-environment interaction predicts the opposite.

To model genotype-environment interaction under the ACE and AE model, we portioned variance due to unique-environmental influences into an intercept (representing environmental variance when *A* = 0) and a slope parameter that is a function of the additive genetic value *A* and represents the interaction effect (henceforth referred to as AxE). This makes the unique-environmental influence different for every individual *j* with additive genetic value ${A}_{j}$ as follows:

1 $${\sigma }_{Ej}^{2}={\text{exp}}({\beta }_{0}+{\beta }_{1} {A}_{j})$$

where ${\beta }_{0}$ denotes the intercept and ${\beta }_{1}$ is a slope parameter that represents AxE. To force the variance to be positive, the exponent was taken (cf. SanCristobal-Gaudy et al. 1998).

Note that the sign of the slope (e.g., ${\beta }_{1}$) determines the direction of the interaction effect and is modelled here as a (log)linear effect. This means that AxE is interpreted as a linear effect (on the log scale), assuming that environmental variance is larger at either a higher (positive) or a lower (negative) level of the genetic value (i.e., larger differences among individuals with similar *A*).

Under the ADE model, the AxE interaction effect (in case of the ADE model referred to as GxE) was conditioned on the *complete* genotype (e.g., both additive genetic and dominant genetic effects) instead of only additive genetic effects. This makes variance due to unique-environmental influences different for every individual *j* with genetic value ${G}_{j}$:

2 $${\sigma }_{Ej}^{2}={\text{exp}}({\beta }_{0}+{\beta }_{1} {G}_{j})$$

where $${G}_{j}$$ represents the genetic value (including both additive and non-additive genetic effects) of individual *j*, $${\beta }_{0}$$ denotes the intercept (when $${G}_{j}=0)$$ and $${\beta }_{1}$$ is a slope parameter that represents GxE.

### Measurement model

Simultaneously to every fitted genetic model, a measurement model in the form of an IRT model was estimated. In IRT models, item scores depend not only on a person’s latent trait (in this case, hyperactivity and inattention respectively) but also on the properties of the items that are contained in the questionnaire such as the difficulty of an item. We used the generalized partial credit model (GPCM), which is an IRT model that is suitable for polytomous, ordinal data (Muraki 1992). The GPCM treats polytomous responses as ordered trait levels and models the probability of endorsing response category *k* over *k—1* for item *i* using both the latent trait value and item parameters. For example, if symptom occurrence is assessed on a scale with 4 answer categories (e.g., “1—*never*”, “2—*sometimes*”, 3—*often* and 4—*always*), we have *K* = 4 and a respondent who selects “*sometimes*” as the response that describes best their situation, is considered to have chosen “*sometimes*” over “*never*” and “*often*” over “*sometimes*”, but to not have chosen “*always*” over “*often*”. This can be interpreted intuitively as if therespondent “passes” all of the preceding ordered response categories before stopping at the final response which reflects most accurately the person’s standing on the latent variable continuum. For each successive response category, the probability of endorsing response category* k* over *k—1* for item *i* is assumed to follow a conditional probability that is dependent on an individual’s latent trait, the discrimination parameter ${\alpha }_{i}$ of that item and thresholds for every item category *k* of item *i*, *β*_*ik*_, that a person has to step through in order to reach the next response category. The discrimination parameter represents how well an item discriminates between the various levels of the latent trait (comparable to a factor-loading in structural equation modelling). Note that by adopting the IRT approach we define ADHD as a latent trait that varies on a continuum, rather than a category of a disorder.

To identify the IRT model, we set the mean of the latent trait as well as the first threshold for all items to zero (i.e.,* μ* = 0 and *β*_*i1*_ = 0).

### One-step estimation of genetic and measurement model

Van den Berg et al. (2007) showed that, in order to take full advantage of the IRT approach, the genetic model and the IRT model have to be fitted concurrently. Only then, the uncertainty of the measurement of the trait is fully taken into account and the spurious finding of a genotype-environment interaction effect due to heterogeneous measurement error can be prevented. Here we use a Bayesian approach to estimate the parameters from the IRT measurement model and the genetic model at the same time, in a unified model. In Bayesian statistical modeling, inference is based on the joint posterior density of all model parameters, which is proportional to the product of a prior probability and the likelihood function of the observed data (Box and Tiao 1992). To obtain this joint posterior density, we applied the Markov chain Monte Carlo (MCMC) algorithm Gibbs sampling (Geman and Geman 1984; Gelfand and Smith 1990). For a detailed description of the specification of the used genetic models in this context, the reader is referred to van den Berg et al. (2006), Schwabe and van den Berg (2014), Schwabe et al. (2015), Schwabe et al. (2017) and Schwabe (2017). For the MCMC estimation, we used the freely obtainable MCMC software package JAGS (Plummer 2003). Statistical details as well as the JAGS syntax for the most complex model (ACE with AxE) can be found in the online Appendix (remaining scripts can be obtained from the first author). For further data handling, the statistical programming language R was used (R Development Core Team 2008). As an interface from R to JAGS, we used the rjags package (Plummer 2013). Note that by applying the IRT approach, we were able to analyze the data on item-level such that the raw data could be used as input in JAGS without having to impute any missing item scores first. JAGS automatically imputes the missing data based on their posterior distribution at every iteration of the algorithm.

To determine which genetic model fitted the data best, while, at the same time, being parsimonious, we fitted all possible combinations of genetic models, with and without genotype-environment interaction (e.g., AE, AE with AxE, ACE, ACE with AxE)^2^ and calculated the deviance information criterion (DIC, Spiegelhalter et al. 2002). The DIC is a measure that estimates the amount of information that is lost when a given model is used to present the data generating process, taking into account both goodness of fit and complexity of the model. All estimated genetic models were fitted simultaneously with a measurement model (IRT model) as described above. For the MCMC estimation, we used the freely obtainable MCMC software package JAGS (Plummer 2003). Note that by applying the IRT approach, we were able to analyze the data on item-level such that the raw data could be used as input in JAGS without having to impute any missing item scores first. JAGS automatically imputes the missing data based on their posterior distribution at every iteration of the algorithm.

For the genetic model that fitted the data best, we calculated the mean and standard deviation for each parameter as well as the 95% highest posterior density (HPD, see e.g. Box and Tiao 1992) interval. The HPD interval can be interpreted as the Bayesian version of a confidence interval (CI) in frequentist statistics. The influence of a model parameter can be regarded as significant when the respective HPD interval does not contain zero (with the exception of the variance components, since these are bounded at zero). Furthermore, in the case that a model that included an interaction effect was chosen as the best-fitting model for the data at hand, we also calculated the effect size of the interaction effect. The effect size was defined as the factor with which the environmental variance component increases for an individual with an additive genetic effect of $A_{i} = \sigma_{a}$. For technical details and power calculations, we refer the reader to Schwabe and van den Berg (2014).

## Results

### Psychometric results

Psychometric analyses showed that the two scales cannot be combined to form one long scale. It was therefore decided to base all further analyses on analysis of SWAN (i.e., the longer scale) item scores only. Results of psychometric analyses furthermore showed where in the distribution of hyperactivity and inattention scores there is the most phenotypic information. With regard to the hyperactivity subscales, it can be concluded that the subscales provide information mostly on individuals in the middle range of hyperactivity (e.g., individuals with neither a very low or a very high degree of hyperactivity). On the other hand, the inattention subscales provide considerable information at the middle range and higher end of the spectrum (i.e., individuals with a high degree of inattention). For the sake of readability, results of psychometric analyses are not described in detail here but can be found in online Appendix C (including item information curves of the combined scales).

### Genetic results

Since the results of the psychometric analyses showed that we cannot combine the two scales, only the items originating from the SWAN questionnaire were used for the genetic analyses. We chose to use the items from the SWAN questionnaire, because (1) the item information curves showed that these items were more informative and (2) the questionnaire contains more items (i.e., 9 hyperactivity items compared to 2 in the SDQ questionnaire, and 9 inattention items compared to 3 in the SDQ questionnaire) and will therefore be more reliable. Since the two MCMC chains of the ADE and ADE with GxE model did not achieve stationarity (i.e., did not approach the joint posterior or target distribution sufficiently closely), the results of these two models are not discussed below, but results are restricted to those based on the remaining genetic models.

#### Hyperactivity

**Fig. 1:**
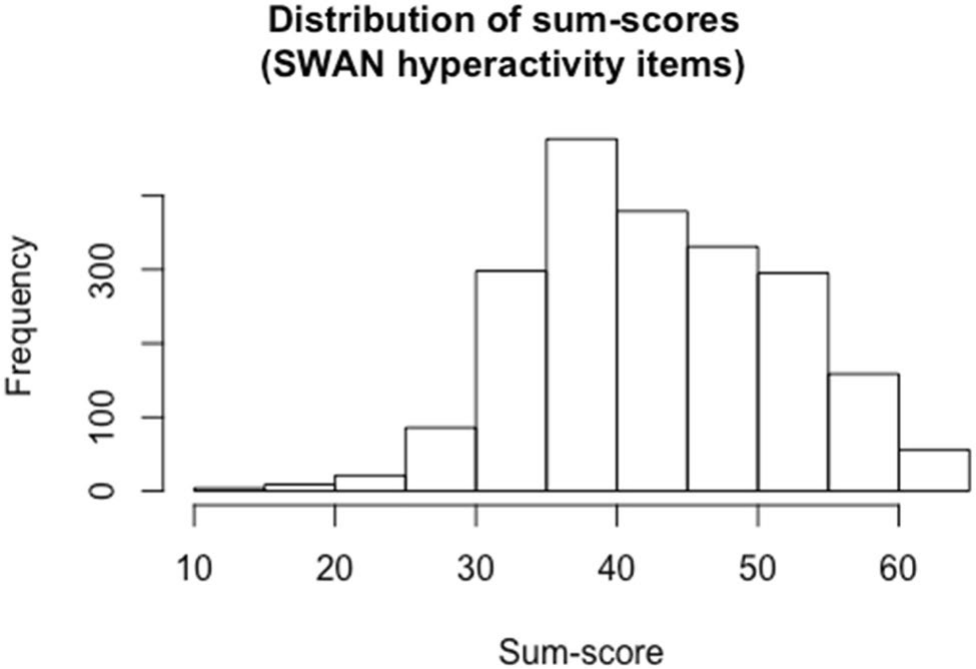
Distribution of the sum-scores of all 9 hyperactivity items (based on all MZ and DZ twins with complete data). A higher sum-score is representative for a higher value on the trait (e.g., a higher degree of hyperactivity)

An item-level analysis of all 9 SWAN questionnaire hyperactivity items resulted in a Cronbach’s alpha equal to 0.89 with a lowest item-rest correlation equal to 0.43. The distribution of sum-scores (based on all MZ and DZ twins without any missing item scores) can be found in Fig. 1. Sum-scores correlated 0.47 among MZ twins and 0.19 among DZ twins.

Table 1 presents the DIC for all fitted genetic models. The AE model with AxE showed the lowest DIC and was therefore chosen as the preferred model for our data.

**Table 1:** Hyperactivity: Model fit (DIC) for all genetic models

Genetic Model	DIC
I. AE	51,755
With AxE	51,658
II. ACE	51,757
With AxE	1,661

*DIC* deviance information criterion

The posterior means and standard deviations of all parameters as well as narrow-sense heritability (${h}^{2},$ defined here as $\frac{{\sigma }_{A}^{2}}{{\sigma }_{A}^{2}+{\text{exp}}({\beta }_{0})}$) resulting from the AE with AxE model can be found in Table 2. Most of the observed variance in hyperactivity could be explained by additive genetic influences, resulting in a narrow-sense heritability equal to 57%. Furthermore, a substantial part of the variance could be explained by unique-environmental influences. Analyses furthermore resulted in a positive and significant AxE interaction, meaning that twins with a high genetic value (e.g., a high value on the latent trait that represents additive genetic influences) tend to show more variance due to unique-environmental influences than twins with lower genetic values. The effect size of this interaction effect was equal to 3.00 which can be regarded as a large effect (see Schwabe and van den Berg, 2014 for technical details).

**Table 2:** Hyperactivity: estimates of all parameters and narrow-sense heritability, based on the AE model with AxE interaction

	Posterior mean (SD)	HPD
${\sigma }_{A}^{2}$	0.25 (0.03)	[0.19;0.31]
${\text{exp}}({\beta }_{0})$	0.19 (0.03)	[0.14;0.24]
${\beta }_{1}$(AxE)	2.20 (0.22)	[1.79;2.65]
${h}^{2}$	0.57 (0.04)	[0.48;0.65]

Total phenotypic variance, defined as ${\sigma }_{A}^{2}+\mathrm{ exp}\left({\beta }_{0}\right)$, was equal to 0.44. HPD refers to the 95% highest posterior density interval and ${h}^{2}$ to narrow-sense heritability

**Fig. 2:**
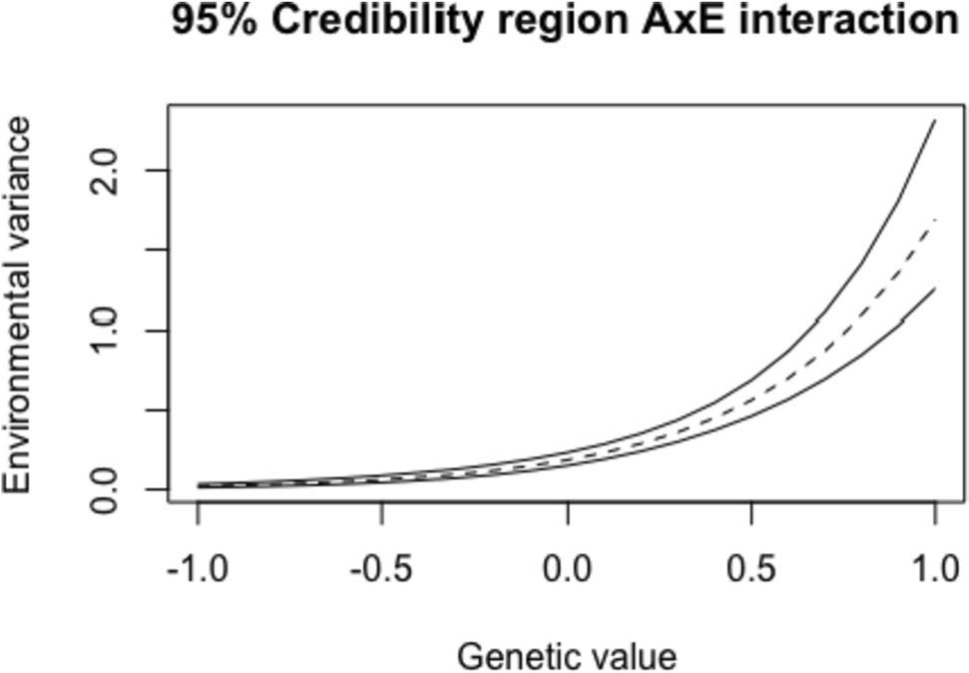
*Hyperactivity*: 95% credibility region of the AxE interaction. The entire range of estimated genetic values (i.e., all posterior means of all twins) is displayed on the *X*-axis with the respective (unstandardized) environmental variance, calculated as ${\sigma }_{Ej}^{2}={\text{exp}}({\beta }_{0}+{\beta }_{1} {A}_{j})$ on the *Y*-axis. A higher genetic value is representative for a higher genetic predisposition for hyperactivity

The 95% credibility region of the AxE interaction effect is displayed for the entire range of estimated genetic values in Fig. 2.

### Inattention

**Fig. 3:**
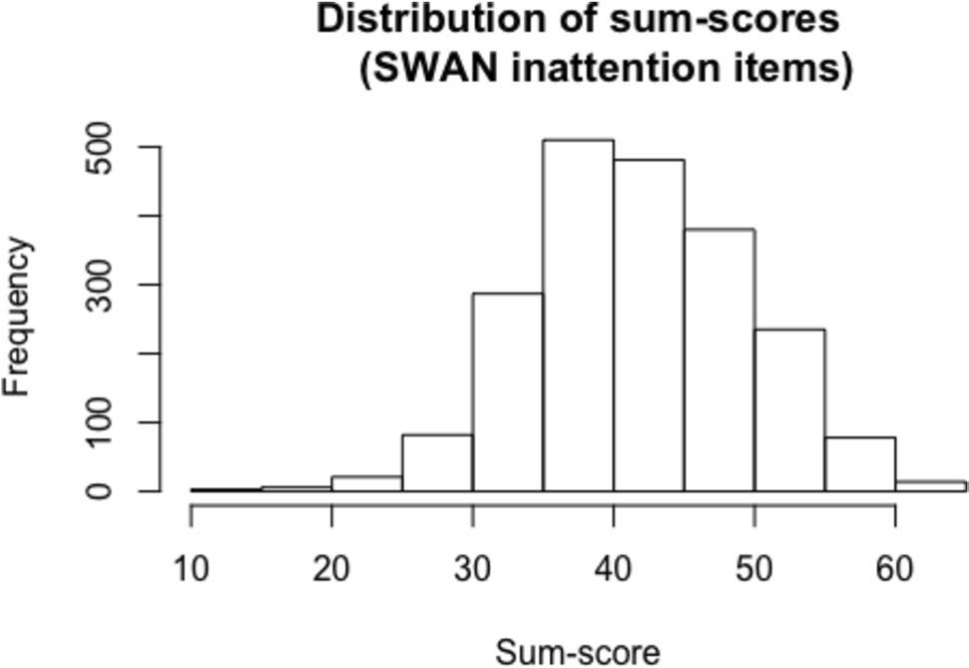
Distribution of the sum-scores of all 9 inattention items (based on all MZ and DZ twins with complete data). A higher sum-score is representative for a higher value on the trait (e.g., a higher degree of inattention)

An item-level analysis of all 9 SWAN questionnaire inattention items resulted in a Cronbach’s alpha equal to 0.89 with lowest item-rest correlation equal to 0.45. The distribution of sum-scores (based on all MZ and DZ twins without any missing item scores) can be found in Fig. 3. Sum-scores correlated 0.46 among MZ twins and 0.24 among DZ twins.

The DIC for all fitted genetic models can be found in Table 3. The ACE model with AxE showed the lowest DIC and was therefore chosen as the preferred model for the twin data.

**Table 3:** Model fit (DIC) for all genetic models

Genetic Model	DIC
I. AE	49,919
With AxE	49,884
II. ACE	49,917
With AxE	49,857

*DIC* deviance information criterion

**Fig. 4:**
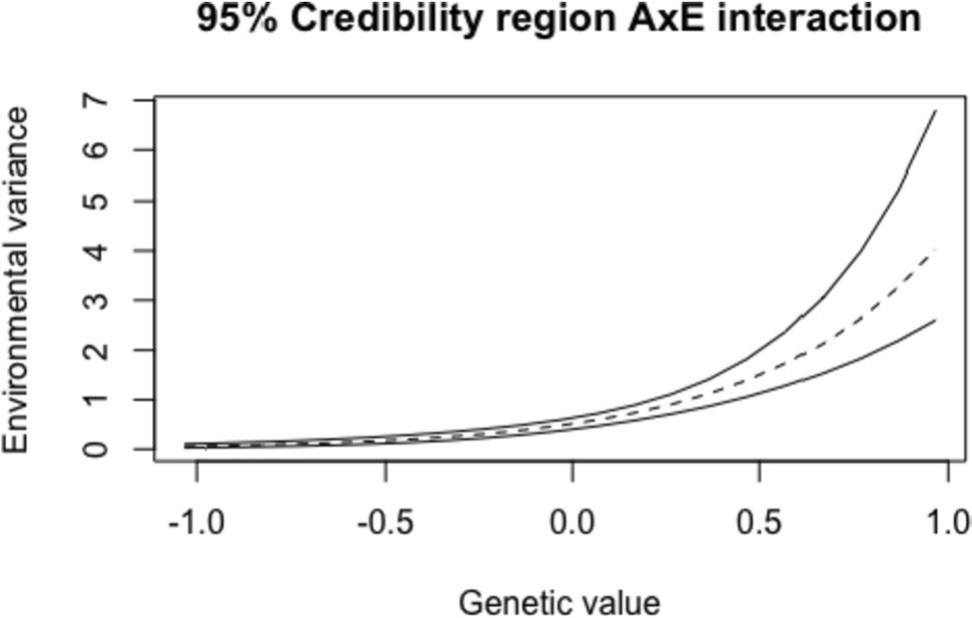
*Inattention*: 95% credibility region of the AxE interaction. The entire range of estimated genetic values (i.e., all posterior means of all twins) is displayed on the *X* axis with the respective (unstandardized) environmental variance, calculated as ${\sigma }_{Ej}^{2}={\text{exp}}({\beta }_{0}+{\beta }_{1} {A}_{j})$ on the *Y* axis. A higher genetic value is representative for a higher genetic predisposition for inattention

Posterior means and standard deviations of all parameters as well as narrow-sense heritability based on the ACE with AxE model can be found in Table 4. In contrast to the results of the hyperactivity scale, unique-environmental influences were the most important source to explain individual differences in inattention, with a narrow-sense heritability equal to 24%. Furthermore, a positive and significant AxE interaction was found, meaning that twins with a high genetic value (e.g., a high value on the latent trait that represents additive genetic influences) tend to show more variance due to unique-environmental influences than twins with lower genetic values. The 95% credibility region of this interaction effect is displayed for the entire range of estimated genetic values in Fig. 4. The effect size of the AxE interaction effect was equal to 3.07 which can be regarded as a large effect (see Schwabe and van den Berg, 2014 for more technical details).

**Table 4:** Inattention: estimates of all parameters and narrow-sense heritability, based on the ACE model with AxE interaction

	Posterior mean (SD)	HPD
${\sigma }_{A}^{2}$	0.27 (0.07)	[0.14;0.41]
${\sigma }_{C}^{2}$	0.31 (0.05)	[0.20;0.41]
${\text{exp}}({\beta }_{0})$	0.51 (0.07)	[0.37;0.65]
${\beta }_{1}$(AxE)	2.16 (0.32)	[1.56;2.79]
${h}^{2}$	0.24 (0.06)	[0.14;0.36]

Total phenotypic variance, defined as ${\sigma }_{A}^{2}+{\sigma }_{C}^{2}+\mathrm{ exp}\left({\beta }_{0}\right)$, was equal to 1.09. HPD refers to the 95% highest posterior density interval and ${h}^{2}$ to narrow-sense heritability

## Discussion

The results of decades of twin studies make it undebatable that ADHD is a heritable disorder. Although research suggests that complex traits are associated with interactions between genetic- and environmental influences, research on genotype-environment interaction using twin modelling has so far not been a focus of genetically informative ADHD studies. Here, we investigated genotype-environment interaction in explaining individual differences in ADHD by analysing the item-level scores of 2168 twins who completed two questionnaires that measure the two ADHD core dimensions, hyperactivity and inattention—the *Strengths and Difficulties Questionnaire* (SDQ) and the *Strength and Weaknesses of ADHD Symptoms and Normal Behavior* (SWAN) questionnaire. Specifically, it was investigated whether a genetic predisposition for hyperactivity (inattention) determines the extent to which unique environmental influences explain variability in hyperactivity (inattention).

Establishing a measure with good psychometric properties is important for finding genomic signals (see van der Sluis et al. 2010; van den Berg and Service 2012; Schwabe et al. 2019). To maximize the psychometric information on ADHD symptoms, here, a thorough psychometric evaluation of the questionnaires was performed to investigate whether the items from the two questionnaires could be combined to form two longer subscales. Multiple analyses suggested, however, that the items from the different questionnaires do not measure the same latent construct and hence cannot be used in the same analysis to reflect the same construct. This is an important result since genomic studies often combine data from multiple cohorts to increase power to find genetic signals (van den Berg et al. 2014). Based on the results of this study, we advise researchers to only use ADHD data from individuals that were assessed by the same questionnaire (either SWAN or SDQ). The psychometric analyses furthermore showed where in the distribution of hyperactivity and inattention scores there is the most phenotypic information. With regard to the hyperactivity subscales, it can be concluded that the subscales provide information mostly on individuals in the middle range of hyperactivity (e.g., individuals who show neither a very low nor a very high degree of hyperactivity). On the other hand, the inattention subscales provide considerable information at the middle range and higher end of the spectrum (i.e., individuals with a high degree of inattention). This is important to note since the power to detect a quantitative trait locus (QTL) does not only depend on variation in genotypes and variation in liability, but also on how well the measurement tool that is used to assess liability discriminates among the genotypes. Improving the scale that is used to measure the phenotype might be much cheaper than ever-increasing sample sizes with the same clinical test (see van den Berg & Service for more details and practical examples).

Based on the results of the psychometric analyses, genetically-informative analyses that included the modeling of genotype-environment interaction were restricted to the items from the SWAN questionnaire. An AE with AxE interaction model based on the hyperactivity data resulted in a moderate narrow-sense heritability (57%), which is comparable to the results of earlier studies (e.g., a meta-analysed narrow-sense heritability equal to 71%, see Nikolas and Burt 2010). Furthermore, while the finding that common-environmental influences contributed a negligible part to the observed variance was also in line with earlier research, our model resulted in a slightly higher estimate of unique-environmental influences. Contrary to earlier research, the results of our ACE with AxE model suggest that the most important source of explaining variance in the inattention dimension were unique-environmental influences while both additive genetic influences as well as common-environmental influences also contributed a significant part to explaining the observed variance. One reason for this seeming contradiction could be that the results of earlier research were based on the analysis of possibly skewed sum-scores whereas all models estimated here were based on latent trait scores.

Next to the models discussed above, also models were estimated that included dominant genetic influences. However, independent of whether we also included a gene-environment interaction, even with a very large number of burn-in iterations (e.g., 75,000), the two MCMC chains did not achieve stationarity (i.e., did not approach the joint posterior or target distribution sufficient closely). Since models were already quite complex and the focus of this study was on a possible gene-environment interaction, we did not estimate any models that included contrast effects but note that these effects might also act on differences among MZ and DZ twins in the degree of hyperactivity and/or inattention.

The most important result of this research is that, on both core dimensions of ADHD, we found significant genotype-environment interaction (AxE), meaning that the unique-environmental variance component is larger in adolescents with a genetic predisposition towards high hyperactivity (inattention) than in adolescents with a genetic predisposition towards low hyperactivity (inattention). Since effect sizes and credibility regions show that these concern large and robust effects, these results contribute to our understanding of the aetiology of ADHD. Note that the mechanism causing this interaction is unknown, meaning that statistically a positive interaction can mean two different things: either unique-environmental factors moderate the influence of additive genetic factors on individual differences in hyperactivity and inattention, or vice-versa (additive genetic factors moderate the influence of unique environments on hyperactivity and inattention). In other words, the degree to which genetic factors contribute to individual differences in hyperactivity may vary depending on exposure to unique environments, and the degree to which unique environmental factors contribute to individual differences in hyperactivity may vary depending on additive genetic factors.

Note that, although the TEDS sample consists of a representative sample of the population in England and Wales (see e.g. Rimfeld et al. 2019) the results discussed above are very specific to this particular population (i.e., 16-year-old twin pairs born between 1994 and 1996) and might not generalize to other (similar) populations. For example, contextual factors might be very different for a sample of twins born in a different time period. Furthermore, the degree of hyperactivity and inattention was assessed using the two subscales of the SWAN which were part of the test booklet sent to twins and answered by themselves (not their parents or teachers). Although the items were derived from the DSM-IV-TR (American Psychiatric Association 2008), research suggests that one needs a more robust assessment (for example a clinical interview and/or neuropsychological assessment) for a more reliable distinction between subjects with and without ADHD (see e.g., Bodenburg et al. 2022).

The method that was used in this research to investigate genotype-environment interaction in ADHD was parametrized such that, both genetic as well as unique-environmental influences, were modelled as latent (i.e., unmeasured) variables. Although this results in great statistical power to determine in an exploratory way whether there is genotype-environment interaction at all (see Schwabe and van den Berg, 2014 for technical details), a drawback is that we do not know what *specific* unique-environmental measures create more individual differences in adolescents genetically predisposed to a high degree of hyperactivity and inattention. Future research on the aetiology of ADHD should focus on the exact nature of the effect found here by collecting specific environmental measures at the individual level. These should specifically be tested in adolescents with many ADHD symptoms, since that is where these environmental factors operate. Given that there is a broad range of influences that can contribute to differences in twin pairs (ranging from subtle prenatal differences to different perceptions of the environment), future research should first focus on variables that have proven to be important for ADHD, like parental stress (Tzang et al. 2008) and adverse childhood experiences like socioeconomic hardship or neighbourhood violence (Brown et al. 2014).

### Supplementary information

Below is the link to the electronic supplementary material.

Supplementary file1 (DOCX 106 kb)

## Footnotes

1 Since correlations (based on sum-scores) in ADHD scores were similar, it was decided to analyse DZ opposite-sex twins together with same-sex DZ twins.

2 Both ADE and ADE with GxE models did not achieve stationarity (did not approach the joint posterior distribution sufficiently closely) and are therefore not discussed (see Appendix B for more details).
