# Supplementary material for: Genotype-Environment Interaction in ADHD: Genetic Predisposition Determines the Extent to Which Environmental Influences Explain Variability in the Symptom Dimensions Hyperactivity and Inattention
Source: Behav Genet. 2024 Jan 25;54(2):169–80. doi: 10.1007/s10519-023-10168-5 (PMC10861382; doi:10.1007/s10519-023-10168-5)
Supplement: Supplementary file 1 — Supplementary file1 (DOCX 106 kb) [file 10519_2023_10168_MOESM1_ESM.docx]

**Appendices:**

**Appendix A.**

The Inattention & Hyperactivity subscales of the SWAN (Swanson, 1981) and SDQ questionnaires (Goodman, 1997). The items were part of the test booklet that was sent to twins at the age of 16 and answered by themselves.

**SWAN**

*Inattention*

1. Give close attention to detail and avoid careless mistakes

2. Sustain attention on tasks or play activities

3. Listen when spoken to directly

4. Follow through on instructions and finish school work or chores

5. Organise tasks and activities

6. Engage in tasks that require sustained mental effort

7. Keep track of things necessary for activities

8. Ignore extraneous stimuli

9. Remember daily activities

*Hyperactive/Impulsive*

1. Sit still (control movement of hands or feet or control squirming)

2. Stay seated (when required by class rules or social conventions)

3. Modulate motor activity (inhibit inappropriate running or climbing)

4. Play quietly (keep noise level reasonable)

5. Settle down and rest (control constant activity)

6. Modulate verbal activity (control excessive talking)

7. Reflect on questions (control blurting out answers)

8. Await turn (stand in line and take turns)

9. Enter into conversations & games without interrupting or intruding

**SDQ**

*Hyperactive/Inattention*

1. I am restless

2. I am constantly fidgeting

3. I am easily distracted

4. I think before I do things

5. I finish the work I am doing

**Appendix B.**

**MCMC estimation in JAGS**

After a burn-in phase of 25,000 iterations for each separate Markov chain, the characterization of the posterior distribution was based on a total of 30,000 iterations from two separate Markov chains. After inspection of convergence plots, it was decided to increase the burn-in period for the ACE with AxE and AE with AxE models for the inattention subscale data to a total of 45,000 iterations, and all models showed convergence after this increase of burn-in iterations. However, even with a very large number of burn-in iterations (i.e., 75,000), the two MCMC chains of both, the ADE and ADE with GxE model, did not achieve stationarity (i.e., did not approach the joint posterior or target distribution sufficiently closely). Convergence plots of all estimated models are not displayed here to save space but can be obtained from the first author.

**JAGS syntax for ACE with AxE model**

model

{

#MZ twins

for (i in 1:NMZ){

C[i] ~ dnorm(0, tau.c)

F[i] ~ dnorm(C[i],tau.a)

A[i] <- F[i]-C[i]

tau.emz[i] <- 1/ exp(beta0+beta1*A[i])

for (twin in 1:2){

mz[i,twin] ~ dnorm(F[i], tau.emz[i]) # latent phenotypes

}

for (j in 1:n.items){

for (k in 1:7){

eta[i , j , k] <- alpha [j] * ( mz[i,1] - beta [j , k])

psum [i , j , k] <- sum(eta[i , j , 1: k])

exp.psum [i , j , k] <- exp( psum [i , j , k])

prob [i , j , k] <- exp.psum [i , j , k] / sum(exp.psum [i , j , 1: 7])

} }

for (j in (n.items+1):(2*n.items)){

for (k in 1:7){

eta[i , j , k] <- alpha [j-n.items] * ( mz[i,2] - beta [j-n.items , k])

psum [i , j , k] <- sum(eta[i , j , 1: k])

exp.psum [i , j , k] <- exp( psum [i , j , k])

prob [i , j , k] <- exp.psum [i , j , k] / sum(exp.psum [i , j , 1: 7])

} }

for (j in 1:(2*n.items)){

Ymz[i , j] ~ dcat ( prob [i , j , 1: 7]) # multinomial distr of data

}

} # end MZ twins

#DZ twins

for (i in 1:NDZ){

Cdz[i] ~ dnorm(0, tau.c)

F1[i] ~ dnorm(Cdz[i], double.tau.a)

F2[i,1] ~ dnorm(F1[i],double.tau.a)

F2[i,2] ~ dnorm(F1[i],double.tau.a)

A2[i,1] <- F2[i,1]-Cdz[i]

A2[i,2] <- F2[i,2]-Cdz[i]

tau.edz[i,1] <- 1/ exp(beta0+beta1*A2[i,1])

tau.edz[i,2] <- 1/ exp(beta0+beta1*A2[i,2])

for (twin in 1:2){

dz[i,twin] ~ dnorm(F2[i,twin], tau.edz[i,twin]) # latent phenotypes

}

for (j in 1:(2*n.items)){

Ydz[i , j] ~ dcat ( probdz [i , j , 1: 7]) # multinomial distr of data

}

for (j in 1:n.items){

for (k in 1:7){

etadz[i , j , k] <- alpha [j] * ( dz[i,1] - beta [j , k])

psumdz [i , j , k] <- sum(etadz[i , j , 1: k])

exp.psumdz [i , j , k] <- exp( psumdz [i , j , k])

probdz [i , j , k] <- exp.psumdz [i , j , k] / sum(exp.psumdz [i , j , 1: 7])

} }

for (j in (n.items+1):(2*n.items)){

for (k in 1:7){

etadz[i , j , k] <- alpha [j-n.items] * ( dz[i,2] - beta [j-n.items , k])

psumdz [i , j , k] <- sum(etadz[i , j , 1: k])

exp.psumdz [i , j , k] <- exp( psumdz [i , j , k])

probdz [i , j , k] <- exp.psumdz [i , j , k] / sum(exp.psumdz [i , j , 1: 7])

} }

}

#Alpha: Set item 1 to 1 to identify the scale,

alpha[1] <- 1

#for the rest of the alpha parameters:

#lognormal prior with expectation of 1 and variance of 10

for (j in 2:n.items){

alpha[j] ~ dlnorm(1, .1)

}

#Beta parameters: Use normal distribution with expectation of 0 and

#variance of 10:

for (j in 1:n.items){

beta [j , 1] <- 0.0

for (k in 2:7){

beta [j , k] ~ dnorm (0, .1)

}

}

tau.a ~ dgamma(1,1)

tau.c ~ dgamma(1,1)

double.tau.a <- 2*tau.a

beta0 ~ dnorm(-1,0.5)

beta1 ~ dnorm(0,.1)

}

**Appendix C.**

**Psychometric results**

*Hyperactivity*

Based on a comparison of different IRT models (e.g., partial credit model (PCM), generalized partial credit model (GPCM) and graded response model (GRM)), the GPCM was selected as the best fitting model for the data at hand and used to investigate item information curves and item fit.

The item information curves that resulted from applying the GPCM to the items of the combined scale (e.g., 9 items from the SWAN questionnaire and 2 items from the SDQ questionnaire) can be found in Figure S1. The item information curves show the amount of information provided by the items taken from the two different questionnaires (SDQ items are displayed with a dotted line in the figure). The Y-axis of the figure depicts the item information and the X-axis shows the theta values where a higher value equals a higher degree of inattention. It can be seen that, overall, the combined scale contains more information on the right side of the trait continuum (e.g., above average to a high degree of hyperactivity). Furthermore, generally, items taken from the SWAN questionnaire are more informative. Lastly, whereas the SWAN questionnaire items contain more information on the extremes of the trait continuum (particularly on twins with a high degree of hyperactivity), items that originate in the SDQ questionnaire discriminate mostly among twins with an average trait value (average hyperactivity).


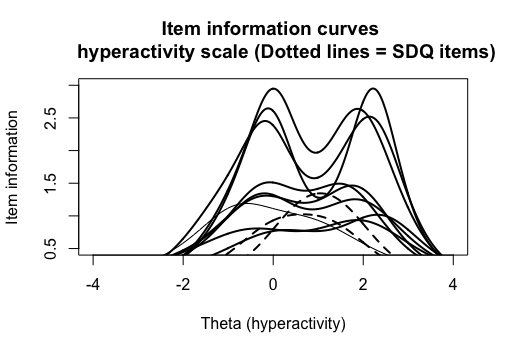


**Figure S1**: Item information curves of the combined hyperactivity scale that contained 9 SWAN items and 2 SDQ (dotted lines) items. The Y-axis shows the item information and the X-axis the estimated theta value (e.g., degree of hyperactivity). A higher theta value is representative for a higher trait value (e.g., a higher degree of hyperactivity).

An analysis of the item fit of all items based on the results of the GPCM of the combined scale showed that the items that were taken from the SDQ questionnaire (“*I am restless*” and “*I am constantly fidgeting*”) resulted in a bad fit, suggesting that there were respondents with answer patterns that badly predicted by the IRT model. For example, there were more respondents with a certain level of hyperactivity that endorsed category 1 than predicted by the GPCM. Although a pattern like that can also be caused by a general misfit of the IRT model, this issue could not be resolved by choosing another model and was therefore interpreted as misfit of the SDQ items within the combined scale. An analysis that was based on solely the 9 SWAN hyperactivity items showed that this model fitted very well and did not result in any item misfit. Statistics and figures that show item fit for every item based on the 9 SWAN hyperactivity items and the combined scales are not displayed here to save space, but can be obtained from the first author.

*Inattention*

The same psychometric analyses were performed for the scale that resulted from combining the inattention items from the SWAN questionnaire (9 items) and the SDQ questionnaire (3 items).

Based on multiple analyses with different IRT models (e.g., partial credit model (PCM), generalized partial credit model (GPCM) and graded response model (GRM)), the GPCM was selected as the best fitting model for the inattention data and used to investigate item information curves of the combined scale as well as item fit of every separate item. The item information curves of all items contained in the combined scale can be found in Figure S2. The item information curves depict the amount of information provided by the items taken from the two questionnaires (items from the SDQ represented by dotted lines). The Y axis of the figure shows the item information, plotted against the theta value (X-axis) where a higher value represents a higher degree of inattention. Similar to the hyperactivity scale, the items that were taken from the SDQ questionnaire (“*I am easily distracted*”, “*I think before I do things*” and “*I finish the work I am doing*”) showed a bad fit. This could not be resolved by applying a different IRT model. A GPCM applied to the SWAN items alone resulted in a good fit. Statistics and figures that show item fit for every item based on the 9 SWAN inattention subscale items and the combined scales are not displayed here to save space, but can be obtained from the first author.


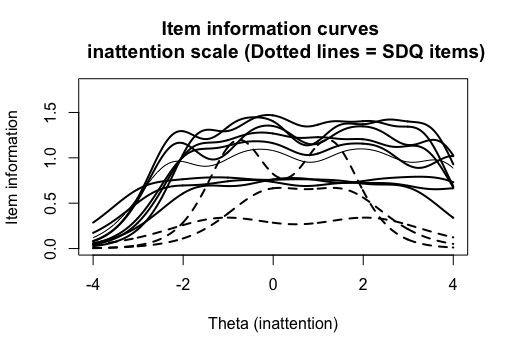


**Figure S2**: Item information curves of the combined inattention scale that contained 9 SWAN items and 3 SDQ (dotted lines) items. A higher theta value is representative for a higher trait value (e.g., a higher degree of inattention).
